# Negative Pressure Wound Therapy in Plastic and Reconstructive Surgery: A Contemporary Narrative Review of Current Evidence and Clinical Applications

**DOI:** 10.7759/cureus.116713

**Published:** 2026-09-22

**Authors:** Daniel Gravino

**Affiliations:** 1 Plastic and Reconstructive Surgery, Mater Dei, Msida, MLT

**Keywords:** closed-incision negative pressure wound therapy, flap reconstruction, negative pressure wound therapy, npwt, plastic surgery, reconstructive surgery, skin graft, wound healing, wound reconstruction

## Abstract

Negative pressure wound therapy (NPWT) is used as an adjunct in the management of complex wounds and reconstructive procedures, including wound-bed management, skin graft fixation, staged reconstruction, donor-site management, and selected closed surgical incisions. The clinical role of NPWT varies according to wound characteristics, reconstructive objectives, and patient factors. This narrative review evaluates the mechanisms of action, clinical applications, complications, safety considerations, cost-effectiveness, and emerging developments associated with NPWT in plastic and reconstructive surgery. Relevant literature addressing NPWT in reconstructive surgery was reviewed, including randomised controlled trials, systematic reviews, meta-analyses, consensus statements, and economic evaluations, with consideration of study design, methodological limitations, clinical applicability, and indication-specific outcomes. NPWT provides mechanical wound stabilisation, controlled exudate removal, reduction of dead space, and graft immobilisation. Its clinical application varies substantially between reconstructive indications, with some applications having a more established role than others. Potential complications include pain, periwound skin injury, bleeding, infection, seal failure, and tissue injury, emphasising the importance of appropriate patient selection and clinical monitoring. NPWT should be considered a reconstructive adjunct with a defined clinical objective rather than a universal wound-healing treatment. Its use should be integrated with appropriate debridement, assessment of perfusion, infection control, protection of critical structures, and timely definitive reconstruction. Further research is required to establish indication-specific treatment strategies and clarify long-term clinical and economic outcomes.

## Introduction and background

Negative pressure wound therapy (NPWT) is a wound-management technique in which a porous wound interface is sealed beneath an occlusive drape and connected to a controlled suction source that applies subatmospheric pressure to the wound or surgical incision [1]. Modern systems permit controlled removal of wound exudate while maintaining a sealed wound environment and can be applied to both open wounds and selected closed surgical incisions.

NPWT has become an established adjunct in plastic and reconstructive surgery, with applications including wound-bed preparation, split-thickness skin-graft fixation, staged extremity reconstruction, burns, donor-site management, selected flap-related wounds, and closed-incision negative pressure wound therapy (ciNPWT) [2-6]. Its clinical use has expanded substantially, but the strength of evidence differs considerably between indications [2-6].

Although several conditions discussed in this review, including diabetic foot wounds and traumatic open fractures, cross traditional specialty boundaries, they are included specifically where NPWT forms part of a reconstructive pathway involving wound-bed preparation, soft-tissue coverage, grafting, flap reconstruction, or limb salvage [2,3]. The focus is therefore not the primary medical or orthopaedic management of these conditions, but the role of NPWT within reconstructive decision-making. Applications without a meaningful reconstructive interface are outside the scope of this review.

The therapeutic rationale for NPWT is based principally on mechanical effects, including macrodeformation, wound stabilisation, exudate evacuation, reduction of dead space, and graft immobilisation [1]. Microdeformation at the wound-interface level may additionally influence cellular proliferation, extracellular-matrix remodelling, and granulation-tissue formation. Proposed biological effects involving angiogenesis, inflammatory signalling, and changes in wound microbiology remain less consistently demonstrated clinically and should be distinguished from established mechanical effects [1].

Recent randomised trials, systematic reviews, meta-analyses, and consensus recommendations increasingly demonstrate that NPWT should not be considered a uniform treatment applicable to all wounds [2-6]. Rather, clinical effectiveness depends upon the indication, wound characteristics, perfusion, reconstructive objective, patient selection, and technical application. This review therefore evaluates not simply whether NPWT is effective, but where it offers clinically meaningful benefit, how it should be applied, and where evidence remains insufficient.

Literature search and evidence prioritisation

This narrative review was informed by a structured literature search of PubMed/MEDLINE, Embase, Scopus, and the Cochrane Library, supplemented by targeted searches of Google Scholar and ClinicalTrials.gov to identify relevant grey literature and ongoing or unpublished trials. Databases were searched from inception to September 2026. Search concepts combined the core terms “negative pressure wound therapy,” “vacuum-assisted closure,” “closed-incision negative pressure wound therapy,” and “negative pressure wound therapy with instillation” with indication-specific terms including “split-thickness skin graft,” “local flap,” “free flap,” “breast reconstruction,” “abdominal donor site,” “burn,” “diabetic foot,” “pressure injury,” “open fracture,” “traumatic wound,” and “chronic wound.” Reference lists of relevant systematic reviews, meta-analyses, consensus statements, and clinical guidelines were also screened to identify additional pertinent studies.

Evidence was prioritised according to methodological strength and relevance to reconstructive practice. Randomised controlled trials, systematic reviews and meta-analyses, including Cochrane reviews, consensus statements, clinical practice guidelines, and large prospective or multicentre cohort studies were assigned the greatest interpretative weight. Observational studies were considered where higher-level evidence was unavailable, particularly for specialised reconstructive applications such as free-flap surgery. Landmark historical and mechanistic studies were retained where necessary to provide technical or biological context, whereas case reports and small uncontrolled series were used only where they contributed information regarding uncommon indications, technical considerations, or rare complications.

Where the available evidence was heterogeneous, conflicting, or of low certainty, this is stated explicitly within the relevant clinical section rather than drawing firm conclusions from lower-level evidence. Experimental and preclinical studies were principally used to evaluate proposed mechanisms of action and were not considered equivalent to clinical evidence of treatment effectiveness.

As this was a narrative rather than a systematic review, no formal Preferred Reporting Items for Systematic Reviews and Meta-Analyses (PRISMA)-based study-selection process, de novo meta-analysis, or study-level risk-of-bias assessment was undertaken. Quantitative effect estimates, confidence intervals, and other statistical outcomes reported in this review were derived from the original randomised trials, systematic reviews, and meta-analyses and are attributed to the corresponding sources. The evidence was synthesised according to clinical indication, methodological quality, consistency of findings, and relevance to contemporary plastic and reconstructive surgical practice.

## Review

Mechanism of action

Established Mechanical Effects

The best-established effects of NPWT are mechanical. Application of subatmospheric pressure produces macrodeformation, approximating wound margins and reducing potential dead space, while continuous fluid evacuation controls exudate and limits local oedema. In reconstructive wounds, these effects stabilise the wound environment and improve contact between a split-thickness skin graft and its recipient bed, thereby limiting shear and fluid accumulation [1-3].

At the wound-interface level, microdeformation generated by the porous dressing alters local tissue geometry. Experimental models demonstrate associated cellular proliferation, migration, and extracellular-matrix remodelling [1-3]. However, the relative contribution of these microscopic effects to patient-level clinical outcomes cannot readily be separated from the mechanical effects of wound stabilisation and fluid evacuation.

Proposed Biological Effects

Changes in angiogenic signalling, inflammatory mediators, cellular differentiation, and local perfusion have been described following NPWT. Experimental studies have demonstrated alterations in vascular endothelial growth factor and other molecular pathways [2,3], but much of this evidence remains preclinical or translational. Such findings support biological plausibility but do not establish that NPWT restores perfusion to ischaemic tissue or independently produces superior clinical healing [3-8]. NPWT should therefore not be considered a substitute for vascular assessment or revascularisation when arterial insufficiency is present.

The microbiological effects are similarly uncertain. Although NPWT facilitates exudate removal, evidence regarding its effect on bacterial burden is inconsistent, and it should not be interpreted as an antimicrobial or biofilm-eradicating therapy [4]. Mature biofilm and established infection continue to require appropriate debridement, source control, and antimicrobial therapy where indicated.

Clinical Interpretation

The clinically relevant mechanisms of NPWT are therefore best understood as wound stabilisation, exudate and dead-space management, reduction of shear, and, in selected applications, graft or incision support [1]. Proposed molecular effects may contribute to healing but remain less firmly established [3-8]. NPWT should consequently optimise the conditions for reconstruction rather than replace adequate debridement, revascularisation, infection control, or definitive soft-tissue coverage.

Overall, the available evidence demonstrates that the clinical effectiveness of NPWT is primarily attributable to its mechanical actions-namely wound stabilisation, dead-space reduction, graft fixation, and exudate management-whereas many proposed molecular mechanisms remain incompletely validated. Consequently, NPWT should be regarded as a reconstructive adjunct that optimises the wound environment rather than a substitute for adequate perfusion, surgical debridement, infection control, or definitive soft-tissue reconstruction. The relative strength of evidence supporting each proposed mechanism is summarised in Table 1.

**Table 1:** Proposed mechanisms of action of negative pressure wound therapy (NPWT), their reconstructive relevance, strength of supporting evidence, and principal limitations. Table created by the authors based on the cited literature.

Mechanism	Reconstructive relevance	Evidence strength	Principal limitation
Macrodeformation	Facilitation of dead space reduction and graft contact	Consistent mechanical effect	Depends on wound geometry
Microdeformation	May stimulate migration and granulation	Experimental and translational	Limited patient-centred evidence
Exudate evacuation	Regulation of fluid dynamics and maceration mitigation	Strong practical rationale	Does not replace source control
Perfusion modulation	May influence local vascular responsiveness	Mainly experimental	Cannot correct major arterial insufficiency
Angiogenic signalling	May support wound-bed maturation	Molecular evidence	Variable clinical efficacy is observed
Graft stabilisation	Reduction of shear stress and fluid accumulation beneath grafts	Consistent reconstructive rationale	Requires a vascular recipient bed

Technical Application and Treatment Parameters

The technical prescription of NPWT should be tailored to the wound, patient, and reconstructive objective rather than applied as a uniform protocol. Although approximately −125 mmHg has historically been widely used, the optimal pressure varies according to indication, tissue characteristics, vascularity, treatment objective, and patient tolerance [7-15]. In diabetic foot wounds without significant vascular compromise, consensus recommendations suggest pressures between approximately −80 and −125 mmHg, whereas lower pressures may be appropriate in the presence of vascular stenosis or occlusive disease [11]. NPWT should therefore be considered an adaptable treatment system rather than a therapy defined by a single pressure setting.

NPWT can be delivered using continuous, intermittent, or variable negative pressure. Continuous therapy provides consistent wound and dressing stabilisation and is particularly relevant where tissue immobilisation is a major objective, including skin-graft fixation and selected reconstructive wounds [16-19]. Intermittent or variable pressure may increase mechanical stimulation of the wound bed, although evidence supporting superiority of one pressure mode across clinical indications remains limited and treatment should therefore be individualised according to wound characteristics, patient comfort, and reconstructive goals [7].

The choice of wound interface should similarly reflect the clinical objective. Foam-based interfaces facilitate transmission of negative pressure throughout the wound and contribute to macro- and microdeformation, exudate evacuation, and wound stabilisation [1,7]. Interface selection should take account of wound geometry, tissue fragility, exposed structures, anticipated granulation, and the risk of tissue adherence. Delicate structures, including exposed vessels, nerves, tendons, vascular grafts, or microvascular pedicles, require appropriate protection before application of negative pressure [20].

Dressing-change frequency should be determined by the wound condition and treatment objective rather than by a universally fixed interval. More frequent reassessment may be required in wounds with infection, heavy exudate, contamination, bleeding risk, or deterioration in tissue viability, whereas graft-bolster applications are generally designed to minimise disturbance during the early period of graft adherence [16-18,20-28]. Treatment should be continued only while NPWT is contributing to a predefined reconstructive endpoint, such as wound-bed preparation, graft stabilisation, control of exudate, or preparation for definitive closure.

Technical parameters may differ substantially according to indication. Skin-graft studies support the use of NPWT as a fixation method because it limits shear and fluid accumulation beneath the graft, although considerable variation exists between studies regarding pressure settings, dressing materials, and treatment duration [16-18]. Consequently, current evidence supports the principle of NPWT-assisted graft fixation more strongly than it supports a single universally optimal pressure protocol.

More specific technical guidance exists for NPWT with instillation and dwell time (NPWTi-d). International consensus recommendations support a negative pressure setting of approximately −125 mmHg for commonly used reticulated open-cell foam systems, with an instillation dwell time of approximately 10 minutes [28]. The duration of the subsequent negative-pressure phase, instillation solution, cycle frequency, and overall treatment duration should nevertheless be adapted to wound characteristics and clinical objectives [28].

Accordingly, the optimal NPWT prescription is indication-specific. Pressure magnitude, mode of therapy, interface material, dressing-change interval, and treatment duration should be selected according to the balance required between wound stabilisation, exudate management, granulation, tissue protection, patient tolerance, and the planned reconstructive endpoint [7,11,16-18,28].

Current Clinical Applications

The contemporary clinical applications of NPWT, together with the highest-quality supporting evidence, principal clinical outcomes, and current recommendations, are summarised in Table 2.

**Table 2:** Summary of the current evidence supporting negative pressure wound therapy across plastic and reconstructive surgery. Table created by the authors based on the cited literature. NPWT, negative pressure wound therapy; NPWTi-d, negative pressure wound therapy with instillation and dwell time; ciNPWT, closed-incision negative pressure wound therapy; RCT, randomised controlled trial; OR, odds ratio; WHiST, Wound Healing in Surgery for Trauma; WOLLF, Wound management of Open Lower Limb Fracture; SSI, surgical site infection

Indication	Evidence	Comparator	Quantitative finding	Certainty/limitation	Practical role
Skin-graft fixation	16-RCT meta-analysis	411 NPWT vs. 401 controls	+8.3% graft take; graft loss OR 0.44; reoperation OR 0.31	Moderate; heterogeneity in protocols	Strongest reconstructive indication
Open fracture	WOLLF RCT	*n* = 460	SSI 7.1% vs. 8.1%; no disability benefit	High-quality RCT; no major benefit	Temporary bridge only
Major trauma incision	WHiST RCT	*n* = 1,548	SSI 5.84% vs. 6.68%, OR 0.87, NS	Large RCT	Not routine
Immediate free-flap NPWT	Systematic review (SR)/meta-analysis	13 studies; 321 flaps	Complications OR 0.34; survival OR 2.34, NS	Mostly retrospective	Highly selective
Free-flap donor site	Meta-analysis	12 studies; 1,074 patients	Dehiscence OR 0.37	Predominantly observational	Consider high-risk sites
Body contouring/panniculectomy	SR/meta-analysis	12 studies	Low dehiscence/reoperation; no clear SSI/seroma benefit	Mixed study designs	Select high-risk patients
NPWTi-d	International consensus	Expert consensus	-125 mmHg; ~10-min dwell	Consensus rather than RCT evidence	Specialist adjunct

NPWT is now used throughout the reconstructive pathway, from temporary wound management to definitive reconstruction. Its greatest clinical value lies in optimising the wound environment through exudate control, reduction of dead space, and tissue stabilisation while definitive reconstructive procedures are planned. Importantly, NPWT should be viewed as an adjunct rather than a substitute for meticulous debridement, vascular assessment, skeletal stabilisation where appropriate, infection control, or timely soft-tissue reconstruction.

Traumatic Wounds and Extremity Reconstruction

In acute traumatic wounds and open fractures, NPWT may provide temporary wound protection following irrigation, debridement, haemostasis, and assessment of exposed structures. Although meta-analytic evidence has suggested reductions in infection and improved wound healing [12], higher-quality randomised evidence is less convincing. A Cochrane review found considerable uncertainty regarding wound healing, infection, pain, quality of life, and closure rates [3], while the Wound management of Open Lower Limb Fractures (WOLLF) economic evaluation demonstrated limited cost-effectiveness, reporting an incremental cost-effectiveness ratio of £267,910 per quality-adjusted life-year (QALY) gained [13]. Similarly, the Wound Healing in Surgery for Trauma (WHiST) economic evaluation demonstrated only minimal gains in QALYs, questioning the routine economic value of incisional NPWT following major lower-limb trauma [4].

Within extremity reconstruction, NPWT may facilitate wound-bed preparation and temporary soft-tissue stabilisation before definitive graft or flap reconstruction. Nevertheless, vascular status, skeletal stability, meticulous debridement, and appropriate timing of reconstruction remain the principal determinants of successful limb salvage [3,12]. Where skin grafting forms part of extremity reconstruction, NPWT may additionally provide graft immobilisation and control of fluid accumulation beneath the graft [16-19].

Burn Surgery

Burn surgery represents another important reconstructive application. Current evidence supports NPWT for skin-graft fixation, wound-bed management, and selected burn wounds. Meta-analyses have demonstrated improved early graft take together with possible reductions in infection, although overall complication rates remain less consistently affected and further high-quality randomised trials are required [14,15]. Accordingly, NPWT should be regarded as an adjunct that optimises wound-bed preparation and graft fixation rather than a replacement for appropriate burn excision, infection surveillance, or definitive wound coverage.

Split-Thickness Skin-Graft Fixation

Among reconstructive indications, split-thickness skin-graft fixation possesses one of the strongest evidence bases. Existing systematic reviews and comparative studies demonstrate improved graft take together with reductions in graft loss, reoperation, seroma formation, and shear-related complications compared with conventional dressings, although reductions in infection are less consistent [16-18].

More recent evidence further strengthens this indication. A 2025 systematic review and meta-analysis of 16 randomised controlled trials included 411 patients treated with NPWT and 401 controls. Compared with conventional dressings, NPWT increased overall graft take by 8.3% (95% confidence interval (CI): 2.97-13.63), increased the odds of successful grafting (odds ratio (OR) 1.86, 95% CI: 1.05-3.30), reduced graft loss (OR 0.44, 95% CI: 0.23-0.85), and reduced reoperation (OR 0.31, 95% CI: 0.13-0.72) [19-20]. A subgroup treated at −80 mmHg demonstrated a 10.0% improvement in graft take (95% CI: 5.69-14.34); however, substantial heterogeneity in the overall graft-take analysis (*I*² = 85%) limits conclusions regarding a universally optimal pressure setting [19].

Taken together, these findings support NPWT as an effective graft bolster, particularly where immobilisation is technically challenging, including irregular surfaces, mobile anatomical regions, and wounds susceptible to shear or fluid accumulation [16-19]. Nevertheless, successful grafting remains dependent upon an adequately vascularised recipient bed, appropriate haemostasis, infection control, and secure graft-to-bed contact; NPWT should not be regarded as capable of compensating for deficiencies in these fundamental reconstructive principles.

Flap and Microsurgical Reconstruction

The role of NPWT in free-flap reconstruction requires particular caution because its potential benefits must be balanced against the need to protect the vascular pedicle and preserve reliable flap monitoring. The available evidence encompasses immediate application of NPWT to viable free flaps as well as management of selected postoperative wound complications [20,21].

A contemporary systematic review and meta-analysis identified 13 studies evaluating immediate postoperative NPWT following free-flap reconstruction, with six studies eligible for quantitative synthesis and 321 free flaps included overall. Compared with conventional wound therapy, NPWT was associated with a reduction in overall postoperative complications (OR 0.34, 95% CI: 0.13-0.88; *n *= 240), but there was no statistically significant improvement in flap survival (OR 2.34, 95% CI: 0.77-7.09; *n *= 252) [21]. The evidence remains limited by heterogeneous treatment protocols and the predominance of non-randomised studies [20,21].

Accordingly, NPWT should not be regarded as a flap-salvage technique. When used around a viable free flap, meticulous protection of the vascular pedicle, perforators, and microvascular anastomoses is essential, and the dressing must not compromise clinical or adjunctive flap monitoring [20,21]. Any suspicion of vascular compromise requires immediate assessment and, where indicated, prompt surgical re-exploration rather than continued NPWT.

The exact effect estimates reported above are derived from the systematic review and meta-analysis by Kawashima and Esmaeili, which included 13 studies and 321 free flaps [20].

Chronic Wounds and Diabetic Foot Reconstruction

Among chronic wounds relevant to reconstructive practice, the evidence is strongest for diabetic foot wounds. Contemporary evidence syntheses demonstrate improved short-term healing outcomes in appropriately selected patients, although successful treatment depends upon adequate perfusion, debridement, infection control, glycaemic management, and effective off-loading [2,11,22]. NPWT should therefore be incorporated within multidisciplinary diabetic foot management rather than used as an isolated wound-healing intervention.

Evidence for other chronic wounds healing by secondary intention is substantially more heterogeneous. A systematic review and meta-analysis of randomised trials suggested potential benefit from NPWT but emphasised limitations arising from heterogeneous wounds, interventions, and generally low-certainty evidence [23]. Consequently, NPWT should be used selectively in chronic wounds where there is a defined wound-management or reconstructive objective rather than on the assumption of universal superiority over conventional wound care.

ciNPWT in Reconstructive Surgery

The evidence supporting ciNPWT is more heterogeneous than that supporting skin-graft fixation. In breast reconstruction, observational studies have reported reductions in infection, wound dehiscence, skin necrosis, seroma formation, and drain duration; however, these findings remain susceptible to selection bias [24-25]. Randomised and meta-analytic evidence has demonstrated less consistent benefit, supporting selective application in patients at increased risk of wound complications rather than routine use in all breast procedures [5,6,24-26].

Similar considerations apply to abdominal donor sites following autologous breast reconstruction. A systematic review and meta-analysis demonstrated a reduction in wound dehiscence with prophylactic ciNPWT, whereas effects on overall wound complications, surgical-site infection, seroma formation, and hospital stay were not statistically significant [27]. Additional clinical evidence similarly supports selective rather than universal application to abdominal donor-site incisions [28].

NPWT With Instillation and Dwell Time

NPWTi-d represents a further evolution of the technology. Integration of instillation and dwell time permits periodic delivery and removal of topical solutions and may facilitate management of selected contaminated, fibrinous, exudative, or anatomically complex wounds following adequate debridement. International consensus recommendations support its selective use after appropriate source control and provide technical guidance regarding pressure, instillation solutions, dwell time, and treatment cycles [29]. Consequently, NPWTi-d should be regarded as a specialist adjunct rather than a replacement for formal surgical debridement or definitive source control.

Specialised Applications and Clinical Integration

Beyond these better-studied indications, NPWT may be considered selectively in anatomically complex, contaminated, or otherwise challenging reconstructive wounds. However, evidence in such specialised settings is frequently derived from observational experience rather than high-quality comparative trials [7,29]. Treatment should therefore be individualised according to wound characteristics, reconstructive objectives, patient tolerance, and the feasibility of adequate clinical surveillance.

Across all indications, NPWT should be integrated into a clearly defined reconstructive strategy with predetermined treatment objectives, regular reassessment, and an established endpoint rather than applied as a stand-alone intervention.

Complications and Contraindications

Although NPWT is generally safe when applied appropriately, complications may occur and are usually related to inappropriate patient selection, inadequate haemostasis, technical errors during dressing application, or insufficient clinical monitoring. Common adverse events include pain during dressing changes, periwound skin injury, maceration, loss of dressing seal, retained foam fragments, bleeding, infection, fistula formation, and tissue ischaemia [26,29,30]. Among these, haemorrhage represents the most serious complication and may be life-threatening, particularly when NPWT is applied over exposed blood vessels, vascular grafts, fresh microvascular anastomoses, or inadequately protected vital structures. Regulatory agencies and international guidelines therefore emphasise meticulous haemostasis, careful protection of vulnerable structures, and ongoing clinical surveillance before and throughout treatment [28,30].

Absolute contraindications include uncontrolled haemorrhage, untreated necrotic tissue with dry eschar, unprotected exposure of major blood vessels, organs, or vascular grafts, and unexplored enteric fistulae. Relative contraindications include severe peripheral arterial disease, therapeutic anticoagulation or coagulopathy, friable skin, active malignancy within the wound, anatomically challenging wounds that prevent an adequate seal, and situations in which appropriate clinical monitoring cannot be ensured [28,30].

Successful application of NPWT depends on careful patient selection, adequate surgical debridement, meticulous haemostasis, appropriate protection of exposed structures, and regular wound reassessment. Increasing pain, unexpected bleeding, malodour, loss of tissue viability, or deterioration in the wound should prompt immediate clinical review and discontinuation of therapy where appropriate. NPWT should therefore be regarded as a treatment requiring active surgical supervision rather than a passive dressing system.

Cost-Effectiveness

The economic value of NPWT varies substantially according to clinical indication, patient risk profile, and healthcare setting. Although NPWT incurs higher initial equipment costs than conventional dressings, these costs may be offset when reductions in wound complications, nursing time, hospital stay, or reoperation are achieved. Consequently, cost-effectiveness should be evaluated according to the specific reconstructive indication rather than assumed universally.

High-quality randomised evidence has questioned the routine economic value of NPWT in traumatic lower-limb wounds. Both the WOLLF and WHiST trials demonstrated minimal gains in QALYs, concluding that routine application for uncomplicated traumatic wounds is unlikely to represent cost-effective use of healthcare resources [3,4]. In contrast, selected high-risk populations-particularly patients with diabetic foot ulcers-may derive greater economic benefit through accelerated wound healing, fewer complications, and reduced resource utilisation [2,31].

Current evidence therefore suggests that NPWT is unlikely to be economically justified for wounds expected to heal predictably with conventional management. Its greatest value appears to lie in carefully selected high-risk wounds where reductions in delayed healing, postoperative complications, or reoperation can offset the increased treatment costs. Ongoing audit of clinical outcomes, complications, and resource utilisation should remain an integral component of NPWT implementation.

Controversies

Despite widespread clinical adoption, several important controversies remain regarding the optimal role of NPWT in reconstructive surgery. The principal debate is no longer whether NPWT exerts beneficial mechanical effects, but rather which patients derive clinically meaningful benefit. Evidence is strongest for split-thickness skin-graft fixation, selected diabetic foot ulcers, and carefully selected burn wounds, whereas benefits following traumatic wounds, free-flap reconstruction, routine ciNPWT, and prophylactic applications remain inconsistent across contemporary studies [6,15-20].

Another unresolved issue concerns the biological effects of NPWT on wound microbiology. Although continuous exudate evacuation may reduce planktonic bacterial burden, convincing evidence that NPWT eradicates mature biofilms remains lacking [9,10,32]. Consequently, NPWT should be regarded as a mechanical adjunct that optimises the wound environment rather than an antimicrobial treatment, and should never replace meticulous debridement, source control, or appropriate antimicrobial therapy.

The role of NPWT in open abdominal management also continues to evolve. Current international consensus recommends NPWT as an effective method of temporary abdominal closure while emphasising that treatment should facilitate early definitive fascial closure rather than unnecessarily prolong open-abdomen management [33]. Future research should therefore focus less on expanding indications and more on identifying the patient populations most likely to achieve meaningful clinical benefit.

Future directions

Future research should prioritise adequately powered multicentre randomised controlled trials using standardised treatment protocols and clinically meaningful outcome measures. Beyond traditional endpoints such as wound closure and graft survival, future studies should incorporate functional recovery, health-related quality of life, limb preservation, reoperation, patient-reported outcomes, and comprehensive cost-effectiveness analyses.

Rapid advances in digital health technologies may further refine NPWT. Pressure and perfusion sensors, remote monitoring platforms, artificial intelligence-assisted wound assessment, machine-learning algorithms, and smart NPWT systems have the potential to improve treatment individualisation and earlier detection of treatment failure. Likewise, combination strategies integrating NPWT with dermal substitutes, biologic dressings, growth factors, stem-cell therapies, three-dimensional scaffolds, or instillation therapy represent promising areas for future investigation. However, prospective clinical validation remains essential before widespread clinical implementation. Ultimately, future innovation should prioritise improved patient selection and demonstrable patient benefit rather than increasing technological complexity alone.

## Conclusions

NPWT is an established adjunct in plastic and reconstructive surgery and can be incorporated into the management of skin-graft fixation, wound-bed preparation, staged reconstruction, donor-site management, selected flap-related wounds, and high-risk surgical incisions. Its principal clinical functions include wound stabilisation, exudate control, reduction of dead space and shear forces, and support of skin-graft fixation. NPWT should be regarded as an adjunct to, rather than a replacement for, fundamental reconstructive principles, including meticulous debridement, assessment of vascularity, infection control, flap monitoring, and timely definitive reconstruction.

The role of NPWT should be determined according to the individual wound, patient characteristics, and intended reconstructive objective. Its application should form part of a comprehensive treatment strategy incorporating adequate perfusion and haemostasis, protection of exposed critical structures, regular clinical reassessment, and a clearly defined treatment endpoint. NPWT should not delay debridement, re-exploration, revascularisation, flap salvage, or definitive reconstruction when these interventions are required. Further investigation is warranted to refine indication-specific treatment strategies, standardise outcome assessment, evaluate longer-term patient outcomes, and establish the economic implications of NPWT across reconstructive practice.
